# Prognostic value of glycemic variability for ICU and in-hospital all-cause mortality in postoperative patients with upper gastrointestinal cancer: a retrospective cohort study

**DOI:** 10.3389/fendo.2026.1898803

**Published:** 2026-09-10

**Authors:** Yilin Chen, Shumin Zhao, Jianguang Xu, Shengshan Xu, Ke-Jie He

**Affiliations:** 1 The Quzhou Affiliated Hospital of Wenzhou Medical University, Quzhou People’s Hospital, Quzhou, Zhejiang, China; 2 The First School of Clinical Medicine, Nanjing University of Chinese Medicine, Nanjing, Jiangsu, China; 3 Department of Thoracic Surgery, Jiangmen Central Hospital, Jiangmen, Guangdong, China

**Keywords:** glycemic variability, mortality, retrospective cohort, upper gastrointestinal cancer, web-based prediction model

## Abstract

**Background:**

Most studies on upper gastrointestinal cancer (UGIC) only focus on static glycemic markers while neglecting glycemic variability (GV). The prognostic role of GV and its subgroup heterogeneity, especially across coronary artery disease (CAD) status, remain unclear. This study aimed to explore the independent predictive value, non-linear pattern and clinical heterogeneity of GV for Intensive Care Unit (ICU) and in-hospital mortality in UGIC postoperative patients.

**Methods:**

This study retrospectively collected and analyzed clinical data from postoperative patients with UGIC at Quzhou People’s Hospital. Survival analyses were employed. They were Kaplan-Meier curves, Cox proportional hazards models, and restricted cubic splines. The study outcomes were 56-day ICU and in-hospital mortality. Subgroup analyses were performed, and three tree-based machine-learning prediction models were implemented, including the random-forest model via the ranger package (version 0.16.0), the XGBoost gradient-boosting model via the XGBoost package (version 1.7.5), and the LightGBM model. Model performance was evaluated and we employed the area under the receiver operating characteristic curve, along with the SHapley Additive exPlanations package, to interpret feature contributions.

**Results:**

Elevated GV independently predicted higher ICU and in-hospital mortality, with spline curves suggesting a potential non-linear-like trend, although formal statistical tests did not confirm a statistically significant nonlinear association. GV and SOFA score were the top prognostic predictors. The GV-mortality association differed between two endpoints and presented obvious CAD-related subgroup heterogeneity. In-hospital mortality better reflected the full prognostic impact of GV, especially in mechanically ventilated and CAD-free patients.

**Conclusion:**

GV is a standalone predictor for ICU and in-hospital all-cause mortality in patients with UGIC, exhibiting a non-linear-like trend and demonstrating robustness across diverse patient populations. Maintaining GV may represent a valuable clinical strategy for improving outcomes in this patient population.

## 1 Introduction

Globally, upper gastrointestinal cancer (UGIC, including esophageal and gastric cancer) is a frequent and related to high rates of morbidity and mortality (1), while esophageal cancer (EC) represents the seventh most prevalent cancer at diagnosis and the sixth leading cause of cancer-related mortality and gastric cancer (GC) is the fifth most common malignancy and the fourth leading cause of cancer-related death worldwide (2, 3). Despite progress in surgical excision, chemotherapy, targeted therapy, and immunotherapy (4, 5), the 5-year overall survival rate remains below 25% for advanced EC and below 30% for advanced GC (6, 7), indicating an extremely poor prognosis primarily attributable to late diagnosis (8), high metastatic potential (9), and treatment resistance (10).

Traditional prognostic factors include tumor stage, histological grade, lymph node metastasis, and performance status (11), whereas metabolic disturbances, particularly glycemic dysregulation (12), are increasingly recognized as key modulators of cancer progression and survival. Diabetes mellitus and stress-induced hyperglycemia are highly prevalent in patients with UGIC. Previous studies have shown that type 2 diabetes and elevated glycemic traits may represent risk factors for the development of UGIC (13, 14). That is because glucose metabolic dysregulation can induce abnormal insulin secretion, and the resultant imbalance in insulin homeostasis is closely associated with energy metabolism, cell proliferation, and inhibition of apoptosis, thereby contributing to the development and progression of malignant tumors and being strongly linked to cancer risk (12), supporting an association between glycemic status and UGIC. However, most clinical and epidemiological studies have focused solely on mean fasting glucose, HbA1c, or binary diabetes status (15), while overlooking glycemic variability (GV), conceptualized as temporal variation of blood glucose levels, calculated by GV (standard deviation of glucose/mean glucose) × 100% (16). Beyond static glycemic indices, GV characterizes real-time dynamic fluctuations of glucose homeostasis in critically-ill conditions. Distinct from single-point or averaged static glucose measurements, GV can reflect the composite biological interplay of physiological stress response, systemic inflammation, and underlying critical-illness severity. Therefore, GV may capture pathological signals that cannot be fully represented by conventional static glycemic biomarkers (17). Accumulating critical-care evidence has compared the prognostic value of diverse glucose-related parameters, including sustained hyperglycemia, hypoglycemic episodes, mean glucose, peak glucose, and dynamic GV (18). Static glucose metrics largely reflect absolute glucose exposure, whereas GV captures glucose instability that cannot be fully represented by conventional static glycemic biomarkers. Clinical evidence further confirms that GV is a critical predictor of adverse prognosis (19, 20). Similar researches have demonstrated that GV is independent factor which linked to mortality in severely ill populations with coronary artery disease (21), sepsis (22), and acute kidney injury (23), with its predictive value even exceeding that of mean glucose levels. These clinical associations may be attributable to circulatory disturbances, endothelial dysfunction, oxidative stress, and systemic inflammation induced by GV (24). However, current clinical guidelines for UGIC have not incorporated GV into risk stratification or personalized management, and the independent prognostic value of GV beyond static glycemic metrics remains to be established. Consequently, there remains an unmet need for evidence-based GV assessment in UGIC care.

To address this gap at the interface of oncology and cancer-associated endocrinology, this study utilized clinical data from Quzhou People’s Hospital, including 689 prognostic patients with UGIC. GV was quantified using the coefficient of variation (CV). The primary outcome was Intensive Care Unit (ICU) all-cause mortality, and in-hospital all-cause mortality was the secondary outcome. To assess the link between GV and mortality risk, we conducted Cox proportional hazards regression and restricted cubic spline (RCS) analyses, and subgroup analyses were used to assess the stability and generalizability of the findings. Furthermore, we used the Boruta algorithm for feature selection, followed by web-based modeling for prediction, and SHapley Additive exPlanations (SHAP) analysis for model interpretability. This study will investigate the independent prognostic impact of GV, a key marker of perturbed glucose homeostasis, on ICU and in-hospital all-cause mortality in UGIC patients, and explore its potential nonlinear dose–response relationship. We hope these real-world observations can add evidence from the cancer-endocrine perspective and inform future efforts to refine risk-stratification frameworks and individualized glycemic management strategies specifically for UGIC postoperative patients.

## 2 Methods

### 2.1 Study design and data sources

An independent retrospective cohort of 689 patients was recruited from The Quzhou Affiliated Hospital of Wenzhou Medical University. Eligible participants were those admitted to the ICU following surgery for UGIC. Baseline characteristics and clinical outcomes were extracted from the institutional electronic health record system. Ethical approval for this retrospective analysis was obtained from the Institutional Review Board of The Quzhou Affiliated Hospital of Wenzhou Medical University (approval No. 2024-129) and was conducted in accordance with the ethical principles of the *Declaration of Helsinki*. Since all data were de-identified and derived from routine clinical practice, the review board fully waived the informed consent requirement.

### 2.2 Patient selection and exclusion

From the database, a total of 689 patients with UGIC who were admitted to the ICU were included in this study. From the initial cohort of patients diagnosed with UGIC, we excluded individuals younger than 18 years old. To ensure the reliability of the GV metrics, patients with an ICU length of stay (LOS) of less than 24 hours or those with fewer than three blood glucose measurements during the first 24 hours of ICU admission were also excluded. Furthermore, patients with significant missing baseline data or those who were not on their first ICU admission were omitted from the final analysis.

### 2.3 Glycemic variability definition and calculation

GV is usually referred to measure variations in glucose or other glucose homeostasis-related parameters over a specified period. In our study, all blood glucose measurements were extracted from the labevents and chartevents tables in our database. Only glucose values recorded within the first 24-hours after admission to the first ICU stay were included for GV calculation. GV was measured via the CV, determined as the SD divided by the mean blood glucose level. This metric reflects the degree of variability relative to the average glucose concentration. The final GV value was obtained by multiplying this ratio by 100% (GV = [SD/mean glucose] × 100%) (16).

To ensure the reliability of the GV metrics, patients with an ICU LOS of less than 24 hours or those with fewer than three blood glucose measurements during the first 24 hours of ICU admission were also excluded. Clinical interventions potentially influencing glucose fluctuations within this 24-hour window, including intravenous or subcutaneous insulin administration, enteral nutrition and parenteral nutrition, were retrieved from the database. These treatment-related covariates were adjusted for in multivariable Cox proportional-hazards regression models to control for confounding effects.

According to GV, the populations were split into two groups according to the 50th (GV = 0.18) percentiles of GV distribution in the cohort. The details are as follows: T1 (≤ 0.18); T2 (> 0.18).

### 2.4 Covariates

Based on clinical relevance and prior literature, the following covariates were considered as potential confounders: age, gender, race, body weight, vital signs (Respiratory rate, Heart rate, MAP, Temperature), comorbidities (COPD, CAD, HF, Hypertension, AFIB, Stroke, T2DM, Renal, Liver, LC), laboratory parameters (WBC count, Hemoglobin, Platelet, pH, PO2, PCO2, Lactate, Creatinine) and therapeutic interventions (MV, RRT, Sedative, Vasopressor). Unless otherwise specified, all covariates were measured within the first 24 hours of ICU admission.

### 2.5 Outcomes

The primary outcome of this study was ICU mortality, defined as all-cause death occurring during ICU hospitalization. A maximum 56-day observation window starting from ICU admission was set, and day 56 was pre-specified as the administrative censoring date. The secondary outcome was in-hospital mortality, defined as all-cause death occurring during the entire hospital stay within this identical 56-day observation window. For all participants, the 56-day observation period uniformly commenced on the day of ICU admission. Patients without a documented death event within the 56-day observation window were censored at day 56.

### 2.6 Statistical analysis

#### 2.6.1 Baseline characteristics

The normality of continuous data was initially assessed. Data following a normal distribution were reported as mean ± SD, with intergroup differences evaluated using the independent samples t-test. For non-normally distributed data, medians with interquartile ranges (25th–75th percentiles) were presented, and the Mann-Whitney U test was used for between-group comparisons. Categorical variables were summarized as counts and percentages [n (%)], and the chi-square test was applied for group comparisons.

#### 2.6.2 Non-linear dose-response analysis

This study employed the RCS model using the rms package to evaluate the non-linear association between GV and the risk of all-cause mortality (25). To guarantee model stability and reduce the risk of overfitting, knots were set at the 10th, 50th, and 90th percentiles of the GV distribution. When the test results indicated a non-linear relationship, a piecewise Cox regression model was further applied to explore the boundary and saturation effects of GV on mortality risk (26).

#### 2.6.3 Survival analysis and regression models

Utilizing survival and survminer package, Kaplan-Meier curves and log-rank tests were used to compare survival distributions across GV groups (27). Cox and logistic regression models were hierarchically constructed to assess the association between GV and all-cause mortality, with results presented as effect sizes and 95% CIs (28). The hazard ratio (HR, from Cox) reflects the effect on instantaneous risk, while the odds ratio (OR, from logistic) reflects the association with the binary outcome.

The specific models were defined as follows. Model 1 used univariable Cox regression, reporting hazard ratios (HR) with 95% confidence intervals (CIs). Models 2–4 employed logistic regression, presenting odds ratios (OR) with 95% CI. Specifically, adjustments included the following. Model 2 was adjusted for all covariates, Model 3 was adjusted for covariates selected by univariable analyses, while Model 4 was adjusted for covariates selected by the Boruta algorithm.

Model 1 preliminarily assessed the crude effect of GV on survival time without adjustment. Models 2–4 used logistic regression for the following reasons. First, ICU mortality and in-hospital mortality were defined as binary endpoints within a constrained 56-day observation window, for which logistic regression offers a straightforward and interpretable analytical framework. Second, logistic regression is less dependent on the proportional-hazards assumption, which is particularly relevant when a large number of covariates are simultaneously adjusted, as in Model 2. Third, Models 2–4 shared an identical logistic-regression framework, enabling direct comparison among three different covariate-selection strategies. Specifically, Model 3 was adjusted for covariates with P < 0.10 identified from univariate analyses. Model 4 was adjusted for covariates selected by the Boruta algorithm.

#### 2.6.4 Subgroup analysis

Subgroup analyses were performed stratified by core clinical characteristics including age, gender, disease severity, and comorbidities. In each subgroup, multivariable Cox regression models adjusted for confounding factors were constructed to evaluate the reliability of the association between GV and all-cause mortality risk across various patients. Interaction analyses were conducted to assess whether the effect sizes differed between subgroups, with an interaction *P*-value < 0.05 considered statistically significant.

#### 2.6.5 Machine learning feature selection and interpretation

To further explore feature importance and support predictive interpretation of risk factors, we performed machine-learning analyses as supplementary sensitivity analyses. First, The Boruta algorithm was employed to perform feature selection among all candidate variables, automatically identifying key variables significantly associated with all-cause mortality risk while eliminating irrelevant and redundant features. Primary prognostic hypothesis testing was implemented via conventional Cox and logistic regression models, while machine-learning approaches were used to explore the relative importance of candidate predictors. Notably, key features highlighted by Boruta, such as disease severity indices, were broadly consistent with statistically significant variables identified in the traditional regression models (including GV and SOFA score). Subsequently, three tree-based ensemble prediction models were built based on the selected feature set: a random forest model implemented via the ranger package (version 0.16.0), and an XGBoost gradient-boosting model implemented via the XGBoost package (version 1.7.5) and a LightGBM model. This analysis is preliminary and exploratory, and no functionally deployable web-based tool for routine clinical application has been developed in the present study. Repeated k-fold cross-validation was adopted for internal validation to tune hyper-parameters and mitigate overfitting risk. It was implemented using the XGBoost package (version 1.7.5). Model performance was assessed using the area under the receiver operating characteristic curve (AUC), and the SHAP package was applied to interpret feature importance (29).

### 2.7 Software and reproducibility

We performed all statistical analyses using R software (version 4.3.1, R Foundation for Statistical Computing) (30). The analysis code and raw data can be obtained from the corresponding author by request.

## 3 Results

### 3.1 Study population baseline characteristics

This study included a total of 689 patients, who were then split into two groups (T1, n=345; T2, n=344) according to the median GV. Baseline characteristics are summarized in **Table 1**.

**Table 1:** Baseline characteristics of original cohort.

	Overall (N = 689)	T1 (N = 345)	T2 (N = 344)	p-value	Missing data (%)
Age	65.05 (12.01)	64.48 (11.92)	65.62 (12.08)	0.25	0.00
Gender (Female)	179 (25.98%)	79 (22.90%)	100 (29.07%)	0.08	0.00
Weight	67.74 (18.99)	70.24 (19.73)	65.24 (17.91)	**<0.01**	0.44
Severity of illness					
SAPS_II	36.87 (13.46)	34.92 (11.60)	38.82 (14.85)	**<0.01**	0.00
SOFA_score	3.78 (3.02)	3.28 (2.39)	4.28 (3.48)	**<0.01**	0.00
Charlson_comorbidity_index	7.04 (2.74)	6.81 (2.77)	7.28 (2.70)	**<0.05**	0.00
Interventions					
MV_therapy (YES)	186 (27.00%)	72 (20.87%)	114 (33.14%)	**<0.001**	0.00
RRT_therapy (YES)	4 (0.58%)	1 (0.29%)	3 (0.87%)	0.61	0.00
Sedative_therapy (YES)	225 (32.66%)	99 (28.70%)	126 (36.63%)	**<0.05**	0.00
Vasopressor_therapy (YES)	139 (20.17%)	49 (14.20%)	90 (26.16%)	**<0.001**	0.00
Comorbidities					
COPD (YES)	97 (14.08%)	47 (13.62%)	50 (14.53%)	0.81	0.00
CAD (YES)	123 (17.85%)	55 (15.94%)	68 (19.77%)	0.23	0.00
HF (YES)	74 (10.74%)	29 (8.41%)	45 (13.08%)	0.06	0.00
Hypertension (YES)	375 (54.43%)	184 (53.33%)	191 (55.52%)	0.62	0.00
AFIB (YES)	216 (31.35%)	111 (32.17%)	105 (30.52%)	0.7	0.00
Stroke (YES)	12 (1.74%)	2 (0.58%)	10 (2.91%)	**<0.05**	0.00
T2DM (YES)	144 (20.90%)	57 (16.52%)	87 (25.29%)	**<0.01**	0.00
Renal (YES)	69 (10.01%)	28 (8.12%)	41 (11.92%)	0.12	0.00
Liver (YES)	18 (2.61%)	5 (1.45%)	13 (3.78%)	0.09	0.00
LC (YES)	23 (3.34%)	7 (2.03%)	16 (4.65%)	0.09	0.00
Vital signs (1st 24 h)					
Respiratory_rate	19.99 (6.97)	19.21 (6.79)	20.77 (7.08)	**<0.01**	0.15
Heart_rate	95.88 (21.37)	94.74 (21.04)	97.03 (21.67)	0.11	0.15
MAP	85.19 (19.71)	85.07 (19.77)	85.31 (19.68)	0.55	0.15
Temperature	36.74 (0.75)	36.80 (0.66)	36.67 (0.83)	**<0.01**	0.44
Laboratory tests (1st 24 h)					
WBC_count	12.26 (8.26)	11.21 (6.64)	13.31 (9.51)	**<0.001**	0.00
Hemoglobin	10.06 (2.00)	10.13 (2.10)	9.98 (1.89)	0.48	0.00
Platelet	233.95 (126.03)	219.67 (111.66)	248.23 (137.62)	<0.05	0.15
pH	7.35 (0.08)	7.36 (0.07)	7.35 (0.09)	0.11	26.71
PO2	126.24 (80.84)	122.39 (69.77)	129.01 (88.02)	0.68	48.04
PCO2	46.47 (13.00)	46.53 (9.53)	46.43 (15.11)	0.22	48.19
Lactate	2.13 (1.51)	1.92 (1.11)	2.32 (1.77)	0.06	25.25
Creatinine	1.00 (0.84)	0.99 (0.99)	1.01 (0.65)	**<0.01**	0.00
Outcome					
ICU_mortality (Death)	63 (9.14%)	14 (4.06%)	49 (14.24%)	**<0.001**	0.00
In-hospital_mortality (Death)	108 (15.67%)	37 (10.72%)	71 (20.64%)	**<0.001**	0.00

Values are presented as mean (standard deviation) for continuous variables and number (percentage) for categorical variables. Variables in bold have p-value < 0.05.

Regarding demographic characteristics, age did not reach statistical significance when comparing the two groups (64.48 ± 11.92 vs. 65.62 ± 12.08, *P* = 0.25). The proportion of female patients was 22.90% and 29.07%, respectively, with no significant difference (*P* = 0.08). Body weight also differed significantly (70.24 ± 19.73 kg vs. 65.24 ± 17.91 kg, *P* < 0.01).

In terms of illness severity, the T2 group had significantly higher SAPS II scores (34.92 ± 11.60 vs. 38.82 ± 14.85, *P* < 0.01), SOFA scores (3.28 ± 2.39 vs. 4.28 ± 3.48, *P* < 0.01), and Charlson Comorbidity Index (6.81 ± 2.77 vs. 7.28 ± 2.70, *P* < 0.05) compared with the T1 group, indicating greater disease severity in the high-GV group.

Regarding interventions, the T2 group showed significantly higher rates of mechanical ventilation (20.87% vs. 33.14%, *P* < 0.001), sedative therapy (28.70% vs. 36.63%, *P* < 0.05), and vasoactive agent use (14.20% vs. 26.16%, *P* < 0.001) than the T1 group, whereas the frequency of renal replacement therapy showed no significant difference between the two groups (0.29% vs. 0.87%, *P* = 0.61).

Comorbidity analysis indicated that the prevalence of T2DM was significantly greater in the T2 group compared with T1 group (16.52% vs. 25.29%, *P* < 0.01), and the prevalence of stroke was also higher (0.58% vs. 2.91%, *P* < 0.05). No marked differences were found for other comorbidities (COPD, CAD, HF, hypertension, atrial fibrillation, renal insufficiency, liver disease, cirrhosis) between the two groups (*P*>0.05).

Regarding vital signs, the T2 group had a significantly higher respiratory rate (19.21 ± 6.79 breaths/min vs. 20.77 ± 7.08 breaths/min, *P* < 0.01) and a significantly lower body temperature (36.80 ± 0.66 °C vs. 36.67 ± 0.83 °C, *P* < 0.01) than the T1 group, while there were no significant differences in heart rate or mean arterial pressure. (*P*>0.05).

Laboratory findings indicated that the T2 group had significantly higher white blood cell counts (11.21 ± 6.64×10^9^/L vs. 13.31 ± 9.51×10^9^/L, *P* < 0.001), platelet counts (219.67 ± 111.66×10^9^/L vs. 248.23 ± 137.62×10^9^/L, *P* < 0.05), and creatinine levels (0.99 ± 0.99 mg/dL vs. 1.01 ± 0.65 mg/dL, *P* < 0.01) compared with the T1 group. There were no significant differences in hemoglobin, pH, PO_2_, PCO_2_, or lactate indices across the two groups (*P*>0.05).

Regarding clinical outcomes, mortality rates differed significantly between GV groups. Overall, ICU mortality was 9.14% and in-hospital mortality was 15.67%. When stratified by GV level, the T1 group had an ICU mortality of 4.06% and an in-hospital mortality of 10.72%, whereas the T2 group had notably higher rates of 14.24% and 20.64%, respectively. Intergroup comparisons showed that both mortality rates were markedly higher in the T2 group than in the T1 group (both *P* < 0.001), suggesting that elevated GV is closely associated with adverse clinical outcomes in patients with UGIC.

In summary, significant differences between the two groups were observed in body weight, disease severity, certain interventions, prevalence of T2DM/stroke, respiratory rate, body temperature, and inflammatory markers. These variables may serve as potential confounders and should be controlled for in the following multivariable analyses.

### 3.2 Non-linear dose-response relationship of GV with mortality

**Figure 1:**
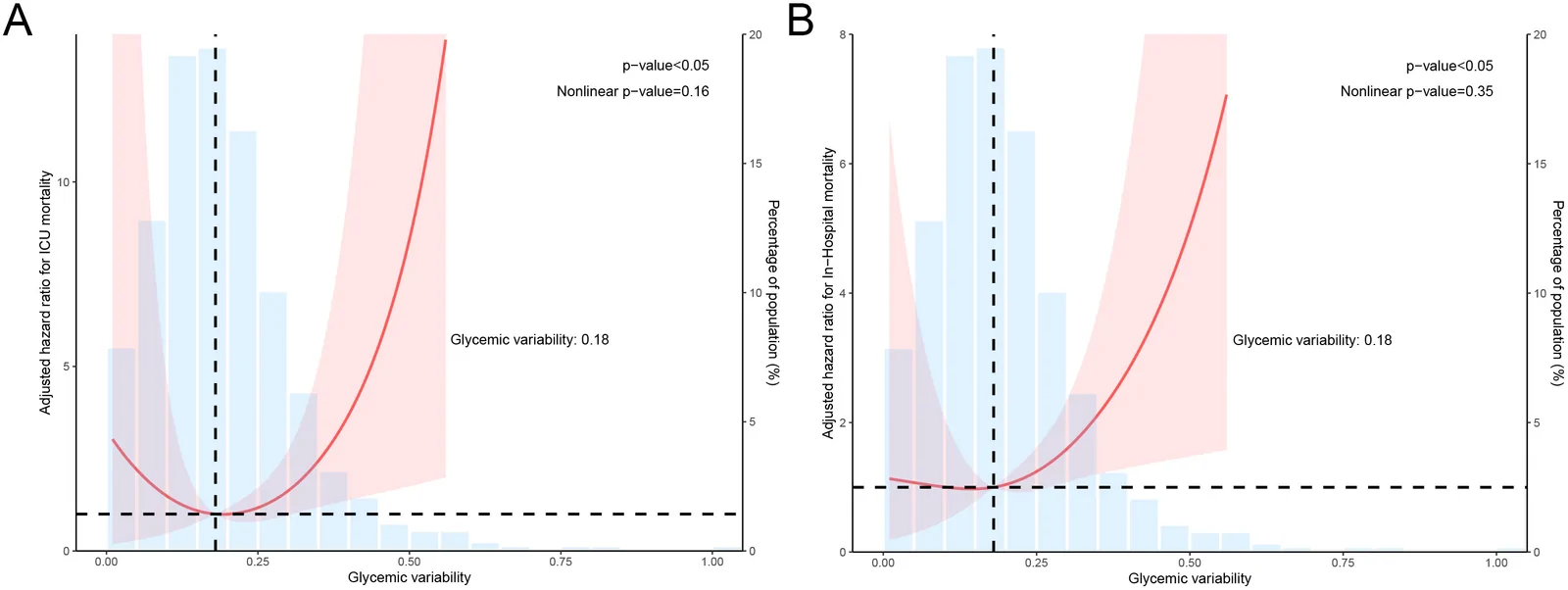
Dose-response relationship for the levels of GV with ICU and in-hospital all-cause mortality measured by restricted cubic spline regression. Spline curves are shown for descriptive visual reference only. Formal tests for nonlinearity yielded *P* = 0.16 **(A)** and *P* = 0.35 **(B)**, indicating no statistically significant departure from linearity. **(A)** Adjusted hazard ratio (HR) for ICU mortality across GV levels in the overall cohort. **(B)** Adjusted hazard ratio (HR) for in-hospital mortality across GV levels in the overall cohort. Shaded areas represent 95% confidence intervals.

To analyze the dose–response relationship between GV and both ICU mortality and in-hospital mortality, this study employed RCS models based on Cox proportional hazards regression after removing the effects of potential confounders (**Figure 1**).

**Figure 2:**
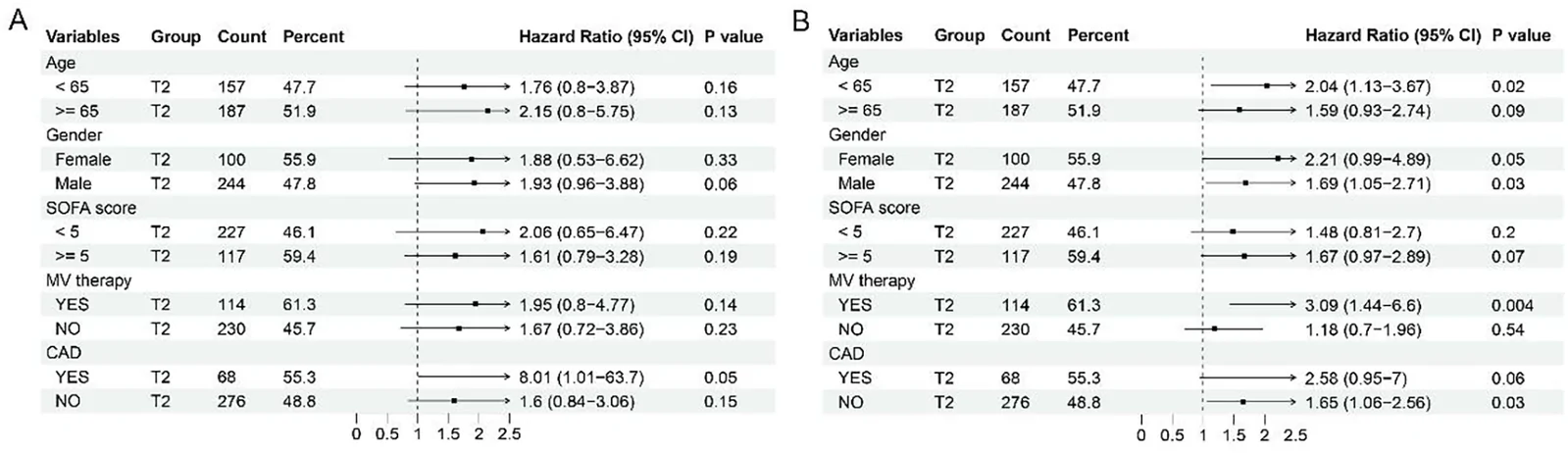
Subgroup analysis of the association between GV and mortality. **(A)** Adjusted HRs and 95% CIs for ICU mortality across GV in subgroups. **(B)** Adjusted HRs and 95% CIs for in-hospital mortality across GV in subgroups.

For ICU mortality (**Figure 2A**), as GV gradually increased from lower levels, the adjusted HR for mortality showed a declining trend, reaching its nadir at GV = 0.18. Beyond this threshold, the risk of death rose sharply with increasing GV, exhibiting an overall U-like patter. The overall association between GV and ICU mortality reached statistical significance (*P* < 0.05), whereas the test for nonlinearity showed no statistical significance (*P* = 0.16). These observations may point toward a potential visual pattern. However, statistical evidence confirming a genuine non-linear association remains limited.

Similar results were observed for in-hospital mortality (**Figure 2B**). The risk of death remained relatively stable when GV was below 0.18, and increased rapidly with GV levels above this cutoff. The overall association between GV and in-hospital mortality was statistically significant (*P* < 0.05), while the nonlinearity test was again not significant (*P* = 0.35). Likewise, a potential non-linear trend could be implied from the curve, yet robust statistical support for a nonlinear association is lacking.

For both outcomes, the curves revealed that the least probability of mortality occurred at a GV level of 0.18, whereas either lower or higher GV scores were related to significantly increased mortality rate. These findings suggest a threshold effect between GV and mortality. Nevertheless, statistical evidence confirming genuine nonlinear or threshold effects was limited given the non-significant nonlinearity tests.

### 3.3 Association between GV and all-cause mortality in various models

**Figure 3:**
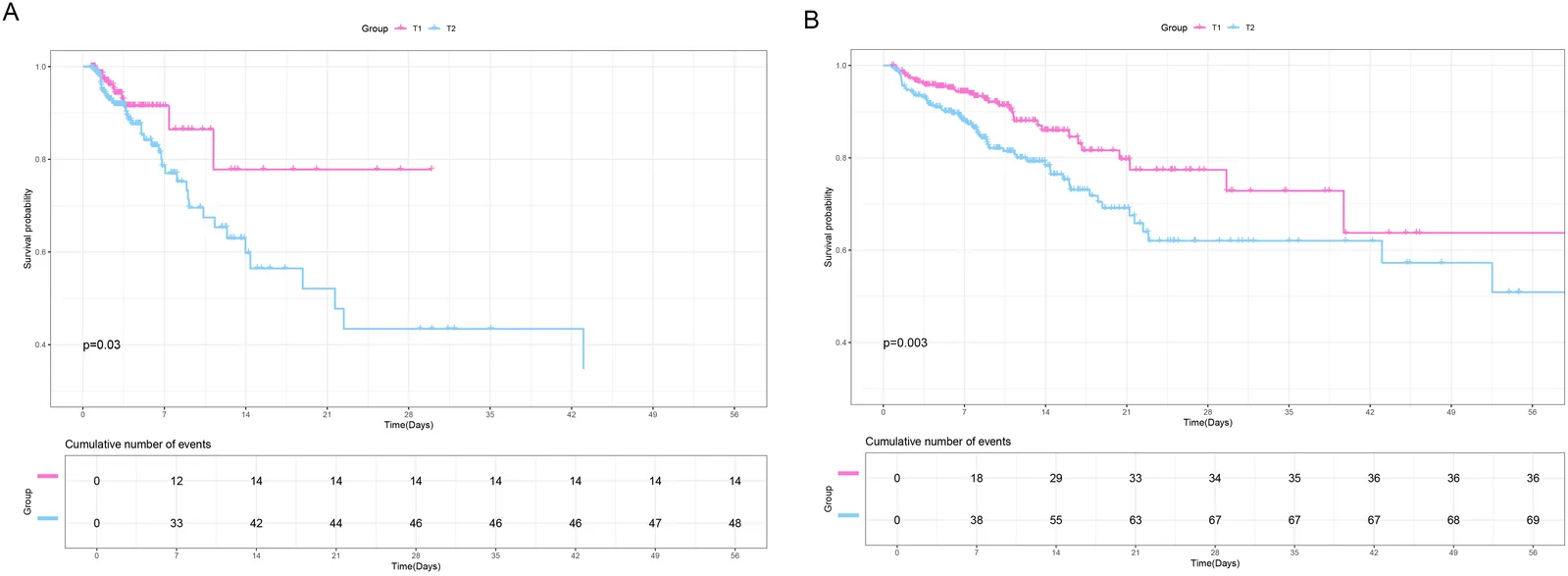
Kaplan-Meier survival curves for all-cause mortality stratified by GV groups. **(A)** Kaplan-Meier curve for 56-day ICU mortality. **(B)** Kaplan-Meier curve for 56-day in-hospital mortality.

To explore the association between GV and all-cause mortality in various models, we used Kaplan–Meier survival analysis to show significant differences in survival curves between the low GV group (T1) and the high GV group (T2) over the 56-day observation period. For ICU mortality (**Figure 3A**), the cumulative survival rate of patients in the low GV group was significantly higher than that in the high GV group (log-rank *P* = 0.03). The survival curve of the high GV group declined more rapidly, indicating that high GV was associated with an increased risk of ICU-period death. And the truncation of the survival curve is due to missing follow-up data after ICU discharge, which limited the complete ascertainment of survival status beyond the ICU stay.

For in-hospital mortality (**Figure 3B**), the difference in survival between the two groups was even more pronounced (log-rank *P* = 0.003). The survival curve of the low GV group remained consistently above that of the high GV group, suggesting a lower risk of in-hospital death in the low GV group. As follow-up time increased, the survival gap between the two groups widened, with the cumulative survival rate of T2 group persistently reduced than that of T1 group. Collectively, these results consistently indicate that high GV is significantly associated with poor prognosis in UGIC, and patients with high GV have significantly higher risks of both ICU and in-hospital mortality than those with low GV.

To confirm the reliability of the findings, we conducted a multi-model analysis (**Table 2**). We first performed univariate Cox regression analysis (Model 1). In the unadjusted model, T2 was significantly related to raised risks of both ICU and in-hospital mortality. Specifically, the HR for ICU mortality was 1.92 (95% confidence interval [CI]: 1.05–3.52, *P* < 0.05), and that for in-hospital mortality was 1.80 (95% CI: 1.21–2.69, *P* < 0.01). These results preliminarily confirmed that GV is a risk factor for death in this cohort, though the associations might be confounded by other clinical variables.

**Table 2:** Primary and secondary outcome analyses with different models for cohort.

In-hospital mortality				ICU mortality	
Model	Group	p-value	Result	p-value	Result
Model 1^1^	T1		1 (Reference)		1 (Reference)
	T2	<0.01	1.8 (1.21, 2.69)	<0.05	1.92 (1.05, 3.52)
Model 2^1^	T1		1 (Reference)		1 (Reference)
	T2	0.05	1.34 (1.09, 1.68)	<0.05	2.53 (1.23, 5.43)
Model 3^1^	T1		1 (Reference)		1 (Reference)
	T2	0.04	1.44 (1.08, 1.86)	<0.01	2.6 (1.31, 5.41)
Model 4^1^	T1		1 (Reference)		1 (Reference)
	T2	0.03	1.45 (1.10, 1.68)	<0.01	2.53 (1.31, 5.12)

Statistical analyses of different models with p-value < 0.05 were displayed in bold.

1OR = Odds Ratio, HR = Hazard Ratio, CI = Confidence Interval.

Model 1: Univariable Cox [HR (95% CI)];

Model 2: Logistic model adjusted with all covariates [OR (95% CI)];

Model 3: Logistic model adjusted with covariates selected by univariable analyses [OR (95% CI)];

Model 4: Logistic model adjusted with covariates selected by Boruta [OR (95% CI)].Note: Hazard ratio (HR) and odds ratio (OR) are not directly comparable. Model 1 estimates instantaneous risk from univariable Cox regression incorporating time to event information; Models 2–4 calculate odds from logistic regression for binary mortality endpoints within the 56 day observation window, without incorporating time to event information.

To eliminate potential confounding effects and validate the independent prognostic value of GV, we constructed and compared multiple multivariable logistic regression models. Model 2 was adjusted for all covariates. After full adjustment, the association between GV and in-hospital mortality remained statistically significant (OR = 1.34, 95% CI: 1.09–1.68, *P* = 0.05); the association with ICU mortality was further confirmed (HR = 2.53, 95% CI: 1.23–5.43, *P* < 0.05). Models 3 and 4 employed variable selection strategies based on univariate analysis and the Boruta algorithm, respectively, to optimize adjustment efficiency. In both optimized models, high GV (T2) consistently emerged as a sole determinant for increased mortality. Specifically, the OR for in-hospital mortality was 1.44 (95% CI: 1.08–1.86, *P* = 0.04) in Model 3 and 1.45 (95% CI: 1.10–1.68, *P* = 0.03) in Model 4. Meanwhile, the associations between GV and ICU mortality remained robust (all *P* < 0.01). **Supplementary Data Sheets 1–8** present detailed results of sensitivity analyses based on univariable Cox regression, multivariable logistic regression, and different variable selection strategies.

### 3.4 Clinical subgroup analysis

Although multivariable regression has confirmed that GV was a sole determinant for death, subgroup analysis was still conducted to confirm the stability of this association across populations with different clinical characteristics, therefore enhancing the robustness and clinical applicability of these outcomes (**Figure 2**).

**Figure 2A** presents the results of univariate Cox regression analysis for ICU mortality. In the high GV group (T2), patients aged ≥65 years, males, those with a SOFA score ≥5, those receiving MV, and those with CAD all showed elevated risks of mortality. Among these, only patients with CAD had a HR approaching statistical significance (HR = 8.01, 95% CI: 1.01–63.7, *P* = 0.05), suggesting an extremely high risk of ICU death in this subgroup. The differences between groups for the other variables (age, gender, SOFA score, MV treatment) showed no statistical significance (all *P* > 0.13).

**Figure 2B** indicates the outcomes of univariate Cox regression assessment for in-hospital mortality. Associations between clinical characteristics and risk were more pronounced. Specifically, age ≥65 years (HR = 1.59, 95% CI: 0.93–2.74, *P* = 0.09), male sex (HR = 1.69, 95% CI: 1.05–2.71, *P* = 0.03), and SOFA score ≥5 (HR = 1.67, 95% CI: 0.97–2.89, *P* = 0.07) all had HRs greater than 1, indicating trends toward increased risk. Patients receiving MV had a significantly elevated risk (HR = 3.09, 95% CI: 1.44–6.6, *P* = 0.004). For CAD patients, although the HR was 2.58, the *P* value of 0.06 did not reach statistical significance. Of note, patients without CAD (NO) exhibited a significantly increased risk of death (HR = 1.65, 95% CI: 1.06–2.56, *P* = 0.03).

### 3.5 Machine learning-based feature selection and model validation

**Figure 4:**
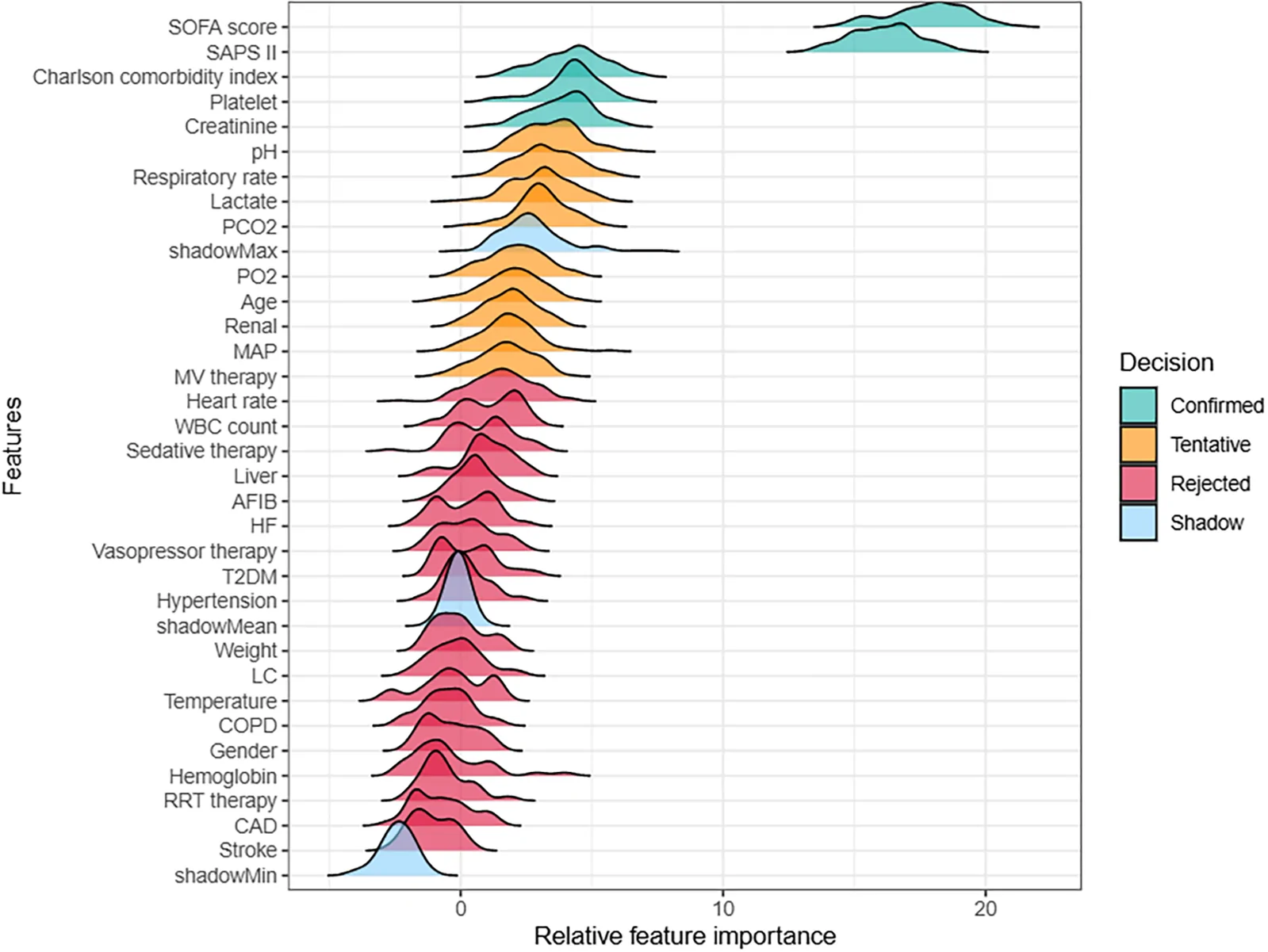
Boruta feature selection between clinically relevant covariates with the primary outcome. Features were categorized into four types: confirmed important features (teal), tentative features (orange), rejected features (red), and shadow features (light blue) serving as random controls. The x-axis represents relative feature importance, while the y-axis displays all features ranked by importance.

To identify clinically meaningful prognostic predictors, feature selection was performed using the Boruta algorithm (**Figure 4**). The results demonstrated that disease severity indicators, including the SOFA score, SAPS II score, and Charlson Comorbidity Index, were identified as highly important variables, with their relative feature importance significantly exceeding that of the shadow features. Some laboratory and physiological parameters, such as platelet count, serum creatinine, PCO_2_, pH value, respiratory rate, lactate, renal function indicators, and PO2, were classified as tentative variables. In contrast, the most of other variables—including age, sex, comorbidities (hypertension, diabetes mellitus, atrial fibrillation), interventions (mechanical ventilation, vasoactive therapy, sedation therapy), body temperature, hemoglobin, and body weight—were deemed unimportant by the algorithm, as their importance did not differ significantly from that of the randomly generated shadow features. In summary, disease severity scores and comorbidity burden emerged as the most robust predictors of mortality outcomes in this cohort, providing a data-driven basis for variable selection in subsequent multivariable regression models.

**Figure 5:**
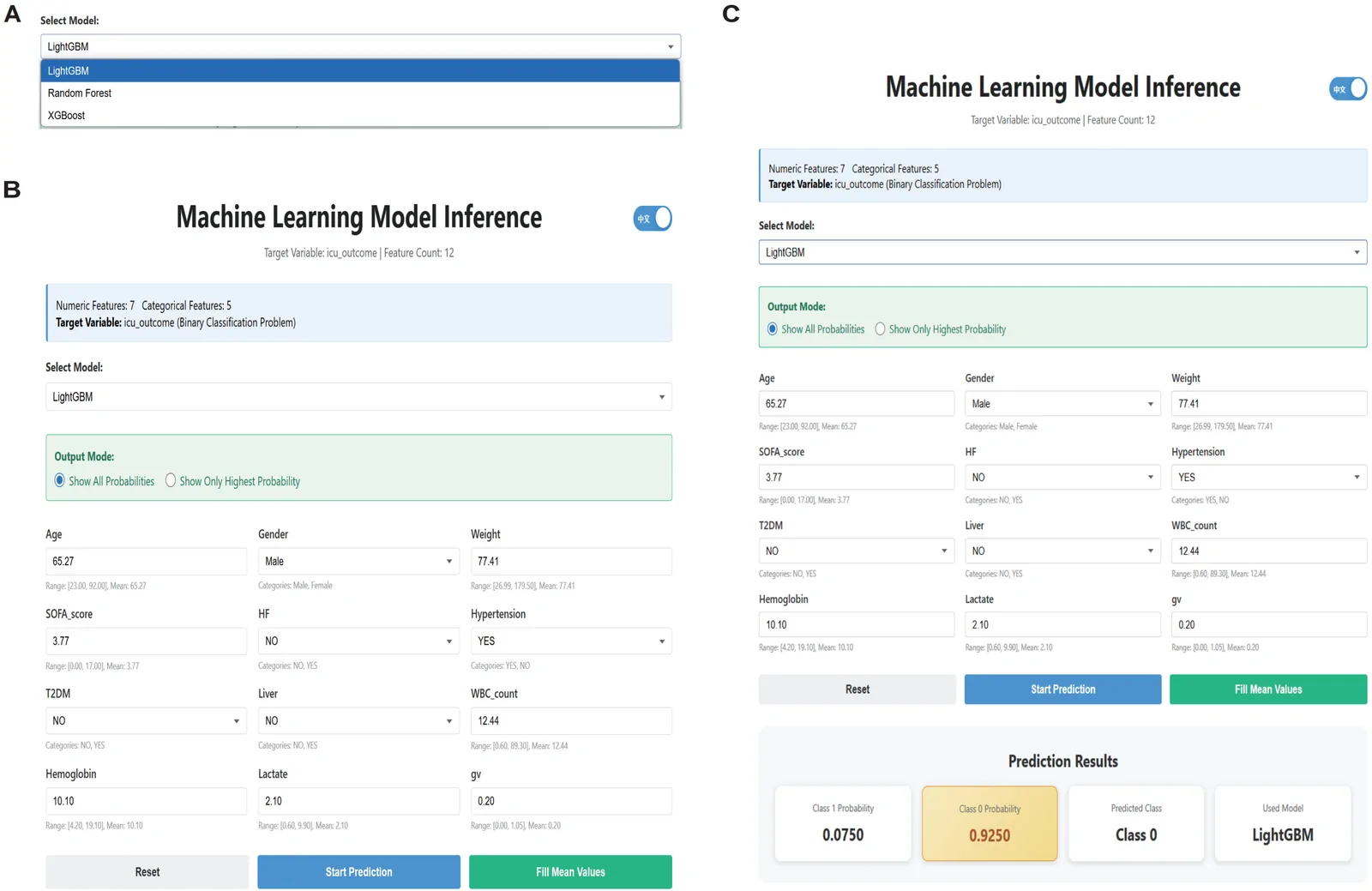
Illustration of the online clinical prediction model interface for individual mortality risk assessment. **(A)** The model‑selection dropdown menu, allowing users to select from LightGBM, Random Forest, and XGBoost. **(B)** The feature‑input panel for 12 patient characteristics, with functional buttons for “Reset” and “Fill‑Mean‑Values”. **(C)** The prediction‑result display panel, which outputs class probabilities after clicking the “Start Prediction” button. This figure illustrates a real‑time prediction example of mortality risk in UGIC patients based on features selected by the Boruta algorithm.

Based on the clinical prediction model interface provided, this study constructed a random forest model for mortality risk prediction using features confirmed by the Boruta algorithm. An example of the model’s application was presented in a real-world clinical setting (**Figure 5**). The interface allows users to select a model (LightGBM, Random Forest, or XGBoost) and input 12 patient characteristics, including Age, Gender, Weight, SOFA score, HF, Hypertension, T2DM, Liver, WBC count, Hemoglobin, Lactate and GV. After clicking “Start Prediction”, the model outputs class probabilities (e.g., Class 1 probability 0.0750, Class 2 probability 0.9250). A reset button and a function to fill mean values are also provided. This interface intuitively demonstrates how the model integrates individual patient features for real-time risk prediction, thereby supporting clinical decision-making. Importantly, **Figure 5** only shows a conceptual schematic interface for illustrative purposes. No functional or deployable web-based prediction tool was developed in the present study, and this figure is merely used to visually demonstrate the workflow of model-driven risk prediction rather than representing a finished clinical product.

**Figure 6:**
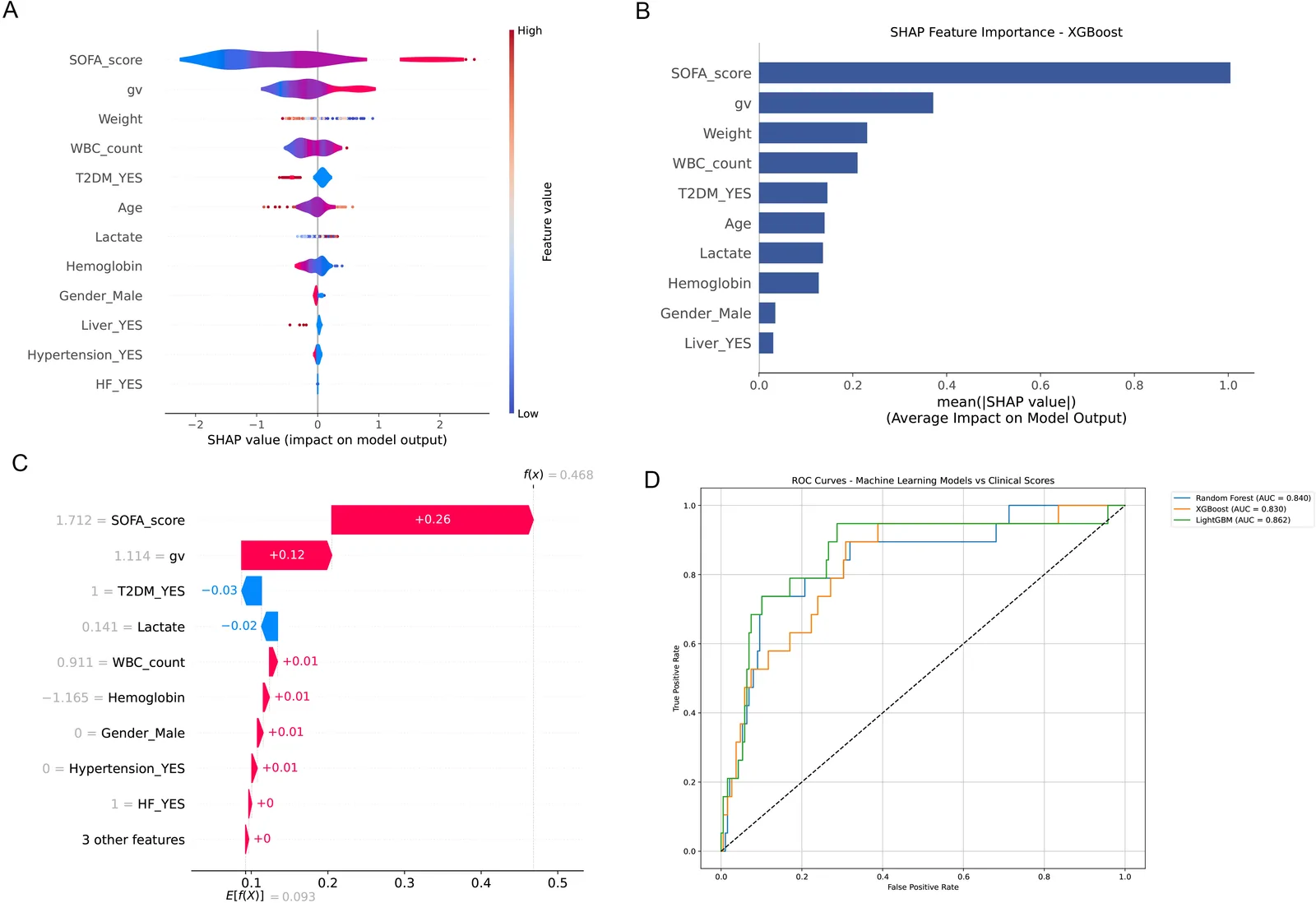
SHAP interpretability analysis and predictive performance of machine learning models for mortality risk prediction. **(A)** Swarm plot showing the distribution of SHAP values for each feature, illustrating the direction and magnitude of their impact on the model output. **(B)** SHAP feature importance ranking based on mean absolute SHAP values. **(C)** SHAP force plot for an individual patient, illustrating the contribution of each feature to the final prediction. **(D)** ROC curves comparing the predictive performance of different machine learning models.

Building on the above framework, the reliability and interpretability of the model were further systematically evaluated. Based on the confirmed features chosen by the Boruta algorithm, this study constructed a random forest model for predicting mortality risk and systematically interpreted the model’s decision logic using SHAP analysis (**Figure 6**). The swarm plot (**Figure 6A**) revealed the direction and magnitude of variable effects: high SOFA score and high GV were associated with positive SHAP values, indicating increased mortality risk, whereas low levels of these variables corresponded to reduced risk. The ranking by mean absolute SHAP value (**Figure 6B**) identified SOFA score as the most influential predictor, followed by GV. The waterfall plot for an individual patient (**Figure 6C**) showed that the predicted mortality risk (0.468) was primarily driven by SOFA score (+0.26) and GV (+0.12), while T2DM and lactate exhibited weak protective effects. Finally, the ROC curves (**Figure 6D**) demonstrated that machine learning models (Random Forest, XGBoost, and LightGBM), achieved excellent discriminative ability (AUC = 0.890, 0.829, and 0.867, respectively), outperforming conventional clinical scoring systems. In summary, SHAP analysis consistently demonstrated that disease severity (SOFA score) and GV are the two most critical factors influencing mortality risk prediction, and their combination significantly improves the prognostic assessment ability for patients with UGIC.

We conducted Boruta feature selection, random-forest modeling and SHAP interpretation as supplementary sensitivity analyses, to explore feature importance complementary to our primary Cox-based hypothesis-testing results. Consistent with the outputs from conventional Cox regression, Boruta identified SOFA score among the most important predictive features. SHAP summary plots further illustrated the direction of risk associations for these key variables. Since machine-learning serves an exploratory purpose in this study, causal inference was mainly derived from the primary Cox regression analyses.

### 3.6 Joint effects of GV and SAPS II score on ICU mortality and length of stay

**Figure 7:**
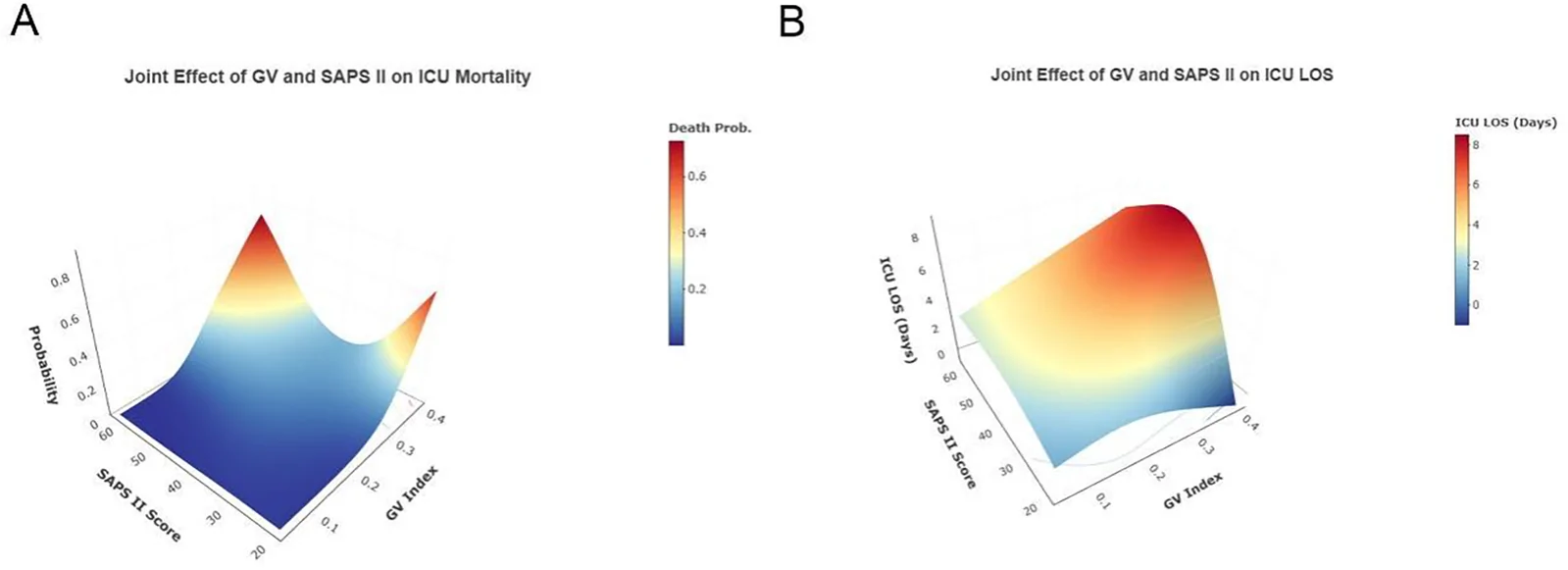
Joint effect of glycemic variability (GV) index and SAPS II score on ICU mortality and ICU length of stay (LOS). **(A)** Surface plot illustrating the predicted probability of ICU mortality across varying levels of GV index and SAPS II score, adjusted for predefined covariates. The color gradient from blue to red represents increasing death probability, ranging from 0 to 0.7.**(B)** Surface plot illustrating the predicted ICU LOS (days) across varying levels of GV index and SAPS II score, adjusted for predefined covariates. The color gradient from blue to red represents increasing ICU LOS, ranging from 0 to 8 days.

To further eliminate the confounding effect of baseline disease severity and visually verify the independent prognostic value of GV, surface plots were applied to visualize the joint and interactive relationship between GV index and SAPS II score on clinical outcomes (**Figure 7**). The joint effect of GV index and SAPS II score on the predicted probability of ICU mortality is presented in **Figure 7A**. After adjusting for other covariates, the surface plot demonstrated that the risk of ICU mortality increased with both higher GV index and higher SAPS II score. Notably, the adverse impact of elevated GV was most pronounced in patients with high SAPS II scores. Among patients with SAPS II scores above the median, the predicted mortality probability rose sharply as GV index increased, reaching a peak in the subgroup with the highest GV and highest SAPS II values. In contrast, for patients with low SAPS II scores (≤ 30), mortality probability remained consistently low regardless of GV index levels. This pattern indicates that the effect of GV on mortality was not simply confounded by disease severity, but rather interacted synergistically with baseline illness severity to exacerbate the risk of death in critically ill patients.

Similarly, the joint effect plot for ICU length of stay (LOS) (**Figure 7B**) revealed that both GV index and SAPS II score were positively associated with prolonged ICU stay. The predicted ICU LOS increased steadily as either variable rose, with the longest stays observed in patients with concurrently high GV index and high SAPS II score. Even among patients with comparable SAPS II scores, those with higher GV index consistently had longer ICU LOS. This finding further supports that GV is not merely a marker of severe illness, but an independent factor contributing to increased healthcare burden in the ICU setting.

## 4 Discussion

Based on the database collected from Quzhou People’s Hospital, the present work was situated at the interface of oncology and cancer-associated endocrinology to systematically investigate the prognostic relevance of GV among patients with UGIC. For the first time, we comprehensively examined this relationship across five dimensions. They were baseline characteristics, non-linear dose–response relationship, multivariable model validation, subgroup analysis, and machine learning, which revealed the potential value of GV in clinical prognostic assessment.

Our core findings confirmed that elevated GV is closely associated with greater disease severity, systemic physiological derangements, and adverse clinical outcomes in patients with UGIC, proposing that GV function as a comprehensive biomarker for early clinical risk stratification. A potential threshold-like pattern for GV-outcome associations, formal statistical tests did not confirm a statistically significant non-linear association or definite threshold effect. After adjusting for potential confounders using multiple statistical models, GV remained an independent risk factor for ICU all-cause mortality, further validating the independence and robustness of this association. Moreover, the relationship was consistently observed across subgroups defined by age, sex, and disease subtypes, indicating good generalizability and stability. However, the results of Boruta variable selection were not completely identical to subgroup analysis outcomes. This could be attributed to the distinct methodological principles. It is because Boruta algorithm screens prognostic variables based on the overall predictive importance in the whole cohort (31), while subgroup analysis explores the heterogeneous effect of GV across different clinical strata (32). Minor inconsistencies between these two approaches are widely recognized and acceptable within prognostic research for cancer-related critical illness.

Notably, the link between GV and mortality differed between the two endpoints of ICU and in-hospital mortality. In the analysis of ICU mortality, no significant subgroup associations were observed, whereas the analysis of in-hospital mortality revealed stronger and more consistent associations, particularly in younger patients, males, those receiving MV, and patients without CAD. This discrepancy was especially prominent in the MV subgroup. Patients on MV showed a significantly elevated risk of in hospital mortality, but this did not reach statistical significance for ICU mortality. This phenomenon may be explained by the longer follow up duration for in-hospital mortality, which captures late deaths occurring after ICU discharge and thus provides higher statistical power to detect associations. For patients receiving MV, who are prone to post ICU complications, using ICU mortality alone may substantially underestimate their true risk of death. These results suggest that in-hospital mortality better reflects the full prognostic impact of GV in critically ill patients.

Interestingly, our subgroup analyses revealed a tentative divergent association pattern between GV and mortality across CAD status among UGIC patients. Among UGIC patients without preexisting CAD, the risk of death was significantly increased (HR = 1.65, 95% CI: 1.06–2.56, *P* = 0.03). Moreover, within the high GV subgroup, the mortality risk diverged markedly between patients with and without CAD. Patients with CAD had higher ICU mortality but relatively lower in-hospital mortality, a pattern that deviates from conventional clinical expectations.

Importantly, this subgroup finding should be interpreted with great caution and is regarded as strictly exploratory. The total number of CAD patients in our cohort was limited (n = 123), and further grouping by GV level resulted in relatively small subgroup sizes, which may lead to unstable risk estimates and wide confidence intervals. Accordingly, the divergent mortality pattern observed in CAD subgroups may be partially attributable to limited sample size and statistical instability, rather than representing a robust clinical or mechanistic difference.

Although the underlying mechanisms remain speculative and cannot be definitively confirmed in the present retrospective cohort, several potential explanations may tentatively interpret this counterintuitive trend. Firstly, the systemic inflammation induced by UGIC itself, the exacerbation of coronary artery disease by inflammation (33), and the amplification of the inflammatory cardiovascular injury cascade by high GV (34). As a malignant proliferative disease, UGIC continuously releases high levels of pro-inflammatory cytokines, including tumor necrosis factor and interleukins, triggering a persistent chronic systemic inflammatory state (33). This chronic inflammation is a key driver of coronary atherosclerosis development and progression. In addition, patients with UGIC often present with malnutrition, immune dysfunction, and oxidative stress imbalance, further aggravating systemic inflammatory burden, continuously damaging vascular endothelial cells, and accelerating the formation, progression, and even rupture of atherosclerotic plaque (35, 36). Consequently, the risk of CAD is much higher in patients with gastrointestinal tumors than in the general population, and higher inflammatory levels are associated with more severe coronary lesions. Importantly, glucose metabolic dysregulation and inflammation are bidirectionally linked (37). High GV activates oxidative stress pathways and exacerbates insulin resistance (38), further provoking the discharge of pro-inflammatory cytokines, leading to a detrimental cycle of “high GV to aggravated inflammation to further glycemic dysregulation”, which continuously exacerbates endothelial injury and coronary lesions (39).

Against this background, patients with different baseline CAD statuses exhibit divergent mortality risk profiles under the combined influence of high GV and inflammation. For UGIC patients with preexisting CAD, the underlying coronary atherosclerosis, reduced coronary blood supply (40), and severely decreased cardiovascular reserve place them in a state of chronic inflammation and myocardial ischemia (41). When high GV is superimposed, acute bursts of inflammation, metabolic disturbances, and hemodynamic fluctuations rapidly induce coronary spasm and plaque instability, worsening myocardial ischemia and hypoxia early during ICU stay, and even triggering acute myocardial infarction, heart failure, circulatory collapse, and other critical cardiovascular events, leading to an obvious increase in short-term ICU mortality (42). However, because these patients have a confirmed diagnosis of CAD, most are on long term regular treatment with statins, beta blockers, and antiplatelet agents. These medications not only regulate lipids and stabilize coronary plaques but also possess anti-inflammatory, endothelium protective, and micro environment modulating effects (43), partially offsetting the acute injury induced by inflammation and high GV. Once patients survive the extreme acute stress of the ICU period, the risk of cardiovascular events rapidly declines, and subsequent in hospital mortality decreases accordingly.

Given the small subgroup sample size, unstable statistical estimates, and unavailable detailed therapeutic information, we emphasize that these subgroup differences are exploratory observations only and cannot be considered conclusive clinical conclusions. Future large-scale prospective studies are required to validate these tentative subgroup phenomena and clarify the underlying mechanisms.

Furthermore, insulin resistance, as a core link connecting high GV (37), inflammation, and tumor progression, further accelerates malignant tumor cell proliferation and inhibits apoptosis (44), while chronic inflammation caused by UGIC exacerbates insulin resistance (45, 46), forming a multiple pathophysiological vicious cycle with high GV, collectively driving adverse clinical outcomes.

Finally, the joint effect surface plot demonstrated that among patients with the same SAPS II score, the risk of ICU mortality increased significantly as GV elevated, further verifying that GV acts as an independent predictor of mortality. Moreover, the adverse effect of GV was more prominent in critically ill patients with higher SAPS II scores, suggesting a synergistic interaction between disease severity and glucose variability on clinical outcomes. These findings further confirm that glucose variability serves as an independent prognostic factor independent of disease severity, and highlight the clinical necessity of minimizing glucose fluctuations in critically ill patients, especially those with severe underlying conditions.

The strengths and core innovations of this study lie not only in the scientific rigor of the analytical methods but also in the systematic and innovative design of the overall research framework. On the methodological front, we leveraged institutional database and performed subgroup analyses, greatly enhancing the reliability of our conclusions. We also incorporated machine learning and model validation: the Boruta algorithm was used to eliminate irrelevant confounding variables, and SHAP analysis was applied to go beyond the limitations of traditional Cox regression and quantitatively assess the contribution of each variable to clinical outcomes. In terms of study design, we constructed a complete research system integrating baseline gradient analysis, nonlinear association exploration, multivariable independent validation, and machine learning interpretability, systematically elucidating the prognostic value of GV in UGIC. This addresses the omission of glycemic dynamics in traditional studies and provides a reference for clinical risk stratification and individualized treatment, substantially enhancing the translational value of our findings.

Nevertheless, several limitations should be acknowledged. First, potential residual confounding remains an important concern. Although we adjusted for key glucose-related covariates including pre-existing diabetes status, insulin therapy, and nutritional management within the initial 24-hour ICU window in multivariable Cox models, residual confounding from unmeasured or incompletely quantified clinical factors cannot be entirely eliminated. Second, the database is a single center medical dataset, and this study did not perform external validation using other databases. The overall sample size was limited, with relatively modest between group differences in outcome events, and the population structure and geographic representation may be insufficient. Therefore, caution is needed when extrapolating our conclusions to clinical populations in different regions or healthcare systems. Third, due to local data security restrictions of our hospital database platform, fitted regression objects, exact lists of covariates selected for Model 3 and Model 4, and model-fit indices such as AIC and BIC cannot be exported for external reporting. Variables entering Model 3 can be inferred from **Table 1** using the univariate screening threshold P<0.10, and Model 4 variables can be inferred from Boruta outputs in **Figure 4**. These inferences cannot replace direct model exports. Model 2 with full-covariate adjustment carries low events-per-variable risk given limited ICU mortality events (n = 63). Our confidence in the independent prognostic effect of GV relies primarily on the consistent association observed across three pre-specified covariate-adjustment strategies. Furthermore, our two machine-learning prediction models were developed on a single-center retrospective cohort and only subjected to internal cross-validation; thus, the risk of overfitting cannot be fully excluded. Calibration analysis, Brier-score evaluation and decision-curve analysis were not performed in the present work. These complementary assessments are needed in future multi-center cohorts with external validation to comprehensively evaluate the clinical utility of these prediction models. Moreover, to maintain statistical power, we excluded cases with incomplete data, further reducing the effective sample size and potentially limiting the generalizability of the conclusions. Additionally, subgroup analyses were confined to a selected set of clinical variables, with limited factors and dimensions examined, making it difficult to fully capture the heterogeneity of the prognostic effect of GV across different populations. Analytically, the median-based dichotomization, while practical for bedside use, may have oversimplified the complex non-linear GV-mortality association identified by RCS analysis. By using the median as the split point, our low-GV group contained patients from both the protective descending limb and the ascending left tail. However, given our aim to test GV as a clinically useful binary risk marker, we considered the median threshold a conservative and interpretable choice. Larger cohorts in the future should employ three-group classification to better characterize the non-linear pattern and determine the optimal clinical cutoff. Further studies are required to address these gaps.

In summary, future cancer-endocrine oriented research should prioritize whether targeted reduction of GV could improve clinical prognosis specifically for UGIC patients. Biomarker assays will help untangle the underlying molecular mechanisms linking tumor-driven inflammation, insulin resistance and glycemic fluctuation. Multi-center prospective cohorts are urgently needed to validate our findings and verify the heterogeneous prognostic performance of GV across diverse UGIC subgroups. Additionally, artificial-intelligence-assisted bedside early-warning tools may be developed to promote standardized application of GV metrics in oncological settings, with the ultimate goal of building evidence-supported glycemic management strategies tailored for postoperative UGIC patients.

## 5 Conclusion

In conclusion, high GV independently predicts increased mortality in critically ill UGIC patients, showing a potential non-linear-like dose–response relationship. GV is a superior prognostic marker to static glucose measures and should be integrated into clinical risk stratification. However, future studies are still required to validate the relation between GV and prognosis in critically ill patients with UGIC and to elucidate the underlying mechanisms.

## Data Availability

The original contributions presented in the study are included in the article/**Supplementary Material**. Further inquiries can be directed to the corresponding author.
