# Supplementary material for: Prognostic value of glycemic variability for ICU and in-hospital all-cause mortality in postoperative patients with upper gastrointestinal cancer: a retrospective cohort study
Source: Front Endocrinol (Lausanne). 2026 Sep 10;17:1898803. doi: 10.3389/fendo.2026.1898803 (PMC13600874; doi:10.3389/fendo.2026.1898803)
Supplement: Supplementary file 1 [file Table1.docx]

Supplementary Table 1. Time Dependent univariable Cox model for ICU mortality

| **Characteristic** | **HR**^1^ | **95% CI**^1^ | **p-value** |
| --- | --- | --- | --- |
| Group |  |  |  |
| T1 | 1.00 | Reference |  |
| T2 | 1.92 | 1.05, 3.52 | 0.03 |
| ^1^HR = Hazard Ratio, CI = Confidence Interval | | | |

Supplementary Table 2. Time Dependent univariable Cox model for In-hospital mortality

| **Characteristic** | **HR**^1^ | **95% CI**^1^ | **p-value** |
| --- | --- | --- | --- |
| Group |  |  |  |
| T1 | 1.00 | Reference |  |
| T2 | 1.80 | 1.21, 2.69 | 0.004 |
| ^1^HR = Hazard Ratio, CI = Confidence Interval | | | |

Supplementary Table 3. Multivariate logistic model adjusted with all covariates for ICU mortality

| **Characteristic** | **OR**^1^ | **95% CI**^1^ | **p-value** |
| --- | --- | --- | --- |
| Group |  |  |  |
| T1 | 1.00 | Reference |  |
| T2 | 2.53 | 1.23, 5.43 | 0.010 |
| Age | 0.98 | 0.95, 1.01 | 0.290 |
| Gender |  |  |  |
| Female | 1.00 | Reference |  |
| Male | 0.73 | 0.34, 1.60 | 0.430 |
| Weight | 0.98 | 0.96, 1.00 | 0.110 |
| SAPS_II | 1.04 | 1.00, 1.08 | 0.050 |
| SOFA_score | 1.24 | 1.06, 1.46 | 0.009 |
| Charlson_comorbidity_index | 0.97 | 0.81, 1.16 | 0.750 |
| MV_therapy |  |  |  |
| YES | 1.00 | Reference |  |
| NO | 1.22 | 0.36, 4.02 | 0.740 |
| RRT_therapy |  |  |  |
| YES | 1.00 | Reference |  |
| NO | 2.61 | 0.09, 127 | 0.600 |
| Sedative_therapy |  |  |  |
| YES | 1.00 | Reference |  |
| NO | 0.68 | 0.23, 2.24 | 0.510 |
| Vasopressor_therapy |  |  |  |
| YES | 1.00 | Reference |  |
| NO | 0.86 | 0.37, 2.11 | 0.740 |
| COPD |  |  |  |
| YES | 1.00 | Reference |  |
| NO | 1.02 | 0.38, 3.07 | 0.980 |
| CAD |  |  |  |
| YES | 1.00 | Reference |  |
| NO | 0.76 | 0.30, 2.01 | 0.560 |
| HF |  |  |  |
| YES | 1.00 | Reference |  |
| NO | 0.60 | 0.21, 1.87 | 0.360 |
| Hypertension |  |  |  |
| YES | 1.00 | Reference |  |
| NO | 0.94 | 0.44, 2.01 | 0.880 |
| AFIB |  |  |  |
| YES | 1.00 | Reference |  |
| NO | 2.22 | 0.96, 5.42 | 0.070 |
| Stroke |  |  |  |
| YES | 1.00 | Reference |  |
| NO | 0.43 | 0.08, 3.67 | 0.370 |
| T2DM |  |  |  |
| YES | 1.00 | Reference |  |
| NO | 2.59 | 0.95, 8.05 | 0.080 |
| Renal |  |  |  |
| YES | 1.00 | Reference |  |
| NO | 1.28 | 0.43, 4.16 | 0.670 |
| Liver |  |  |  |
| YES | 1.00 | Reference |  |
| NO | 25.2 | 0.85, 1,305 | 0.070 |
| LC |  |  |  |
| YES | 1.00 | Reference |  |
| NO | 0.73 | 0.07, 15.2 | 0.810 |
| Respiratory_rate | 1.04 | 0.99, 1.09 | 0.140 |
| Heart_rate | 1.01 | 1.00, 1.03 | 0.130 |
| MAP | 1.00 | 0.99, 1.02 | 0.630 |
| Temperature | 0.82 | 0.52, 1.26 | 0.360 |
| WBC_count | 1.00 | 0.96, 1.04 | 0.990 |
| Hemoglobin | 0.93 | 0.78, 1.10 | 0.420 |
| Platelet | 1.00 | 1.00, 1.00 | 0.600 |
| pH | 3.53 | 0.04, 349 | 0.580 |
| PO2 | 1.00 | 1.00, 1.00 | 0.850 |
| PCO2 | 1.01 | 0.99, 1.04 | 0.250 |
| Lactate | 1.16 | 0.96, 1.40 | 0.120 |
| Creatinine | 1.13 | 0.80, 1.57 | 0.470 |
| ^1^OR = Odds Ratio, CI = Confidence Interval | | | |

Supplementary Table 4. Multivariate logistic model adjusted with covariates selected by uni-variable analyses for ICU mortality

| **Characteristic** | **OR**^1^ | **95% CI**^1^ | **p-value** |
| --- | --- | --- | --- |
| Group |  |  |  |
| T1 | 1.00 | Reference |  |
| T2 | 2.60 | 1.31, 5.41 | 0.008 |
| Weight | 0.98 | 0.96, 1.00 | 0.040 |
| SAPS_II | 1.02 | 0.99, 1.06 | 0.220 |
| SOFA_score | 1.23 | 1.08, 1.42 | 0.002 |
| Charlson_comorbidity_index | 0.99 | 0.85, 1.14 | 0.870 |
| MV_therapy |  |  |  |
| YES | 1.00 | Reference |  |
| NO | 0.98 | 0.31, 2.98 | 0.980 |
| Sedative_therapy |  |  |  |
| YES | 1.00 | Reference |  |
| NO | 0.73 | 0.26, 2.23 | 0.560 |
| Vasopressor_therapy |  |  |  |
| YES | 1.00 | Reference |  |
| NO | 1.07 | 0.50, 2.39 | 0.860 |
| T2DM |  |  |  |
| YES | 1.00 | Reference |  |
| NO | 2.52 | 1.01, 7.09 | 0.060 |
| Respiratory_rate | 1.03 | 0.99, 1.07 | 0.190 |
| Heart_rate | 1.01 | 1.00, 1.03 | 0.120 |
| WBC_count | 1.01 | 0.98, 1.04 | 0.640 |
| Hemoglobin | 0.91 | 0.78, 1.07 | 0.260 |
| pH | 1.08 | 0.03, 45.5 | 0.970 |
| Lactate | 1.08 | 0.90, 1.27 | 0.400 |
| Creatinine | 1.14 | 0.84, 1.50 | 0.360 |
| ^1^OR = Odds Ratio, CI = Confidence Interval | | | |

Supplementary Table 5. Multivariate logistic model adjusted with covariates selected by Boruta algorithm for ICU mortality

| **Characteristic** | **OR**^1^ | **95% CI**^1^ | **p-value** |
| --- | --- | --- | --- |
| Group |  |  |  |
| T1 | 1.00 | Reference |  |
| T2 | 2.53 | 1.31, 5.12 | 0.007 |
| Creatinine | 1.03 | 0.76, 1.33 | 0.82 |
| Platelet | 1.00 | 1.00, 1.00 | 0.65 |
| Charlson_comorbidity_index | 0.95 | 0.83, 1.08 | 0.43 |
| SOFA_score | 1.27 | 1.14, 1.43 | <0.001 |
| SAPS_II | 1.03 | 1.00, 1.07 | 0.04 |
| ^1^OR = Odds Ratio, CI = Confidence Interval | | | |
|  | | | |

Supplementary Table 6. Multivariate logistic model adjusted with all covariates for In-hospital mortality

| **Characteristic** | **OR**^1^ | **95% CI**^1^ | **p-value** |
| --- | --- | --- | --- |
| Group |  |  |  |
| T1 | 1.00 | Reference |  |
| T2 | 1.34 | 1.09, 1.68 | 0.05 |
| Age | 0.99 | 0.97, 1.02 | 0.51 |
| Gender |  |  |  |
| Female | 1.00 | Reference |  |
| Male | 0.83 | 0.47, 1.48 | 0.52 |
| Weight | 0.98 | 0.96, 0.99 | 0.004 |
| SAPS_II | 1.03 | 1.00, 1.07 | 0.06 |
| SOFA_score | 1.25 | 1.10, 1.43 | <0.001 |
| Charlson_comorbidity_index | 1.00 | 0.87, 1.15 | 0.98 |
| MV_therapy |  |  |  |
| YES | 1.00 | Reference |  |
| NO | 0.98 | 0.36, 2.61 | 0.97 |
| RRT_therapy |  |  |  |
| YES | 1.00 | Reference |  |
| NO | 4.09 | 0.16, 204 | 0.45 |
| Sedative_therapy |  |  |  |
| YES | 1.00 | Reference |  |
| NO | 1.28 | 0.53, 3.34 | 0.6 |
| Vasopressor_therapy |  |  |  |
| YES | 1.00 | Reference |  |
| NO | 1.60 | 0.80, 3.33 | 0.2 |
| COPD |  |  |  |
| YES | 1.00 | Reference |  |
| NO | 1.25 | 0.60, 2.79 | 0.57 |
| CAD |  |  |  |
| YES | 1.00 | Reference |  |
| NO | 0.63 | 0.32, 1.24 | 0.17 |
| HF |  |  |  |
| YES | 1.00 | Reference |  |
| NO | 0.70 | 0.31, 1.61 | 0.39 |
| Hypertension |  |  |  |
| YES | 1.00 | Reference |  |
| NO | 1.03 | 0.58, 1.83 | 0.91 |
| AFIB |  |  |  |
| YES | 1.00 | Reference |  |
| NO | 1.29 | 0.70, 2.43 | 0.42 |
| Stroke |  |  |  |
| YES | 1.00 | Reference |  |
| NO | 0.53 | 0.12, 2.97 | 0.43 |
| T2DM |  |  |  |
| YES | 1.00 | Reference |  |
| NO | 1.60 | 0.78, 3.42 | 0.21 |
| Renal |  |  |  |
| YES | 1.00 | Reference |  |
| NO | 1.22 | 0.52, 3.03 | 0.65 |
| Liver |  |  |  |
| YES | 1.00 | Reference |  |
| NO | 4.95 | 0.47, 60.5 | 0.19 |
| LC |  |  |  |
| YES | 1.00 | Reference |  |
| NO | 0.52 | 0.08, 4.19 | 0.51 |
| Respiratory_rate | 1.04 | 1.00, 1.08 | 0.06 |
| Heart_rate | 1.01 | 1.00, 1.03 | 0.04 |
| MAP | 1.00 | 0.99, 1.01 | 0.74 |
| Temperature | 1.11 | 0.79, 1.56 | 0.54 |
| WBC_count | 1.01 | 0.99, 1.04 | 0.33 |
| Hemoglobin | 0.93 | 0.81, 1.06 | 0.25 |
| Platelet | 1.00 | 1.00, 1.00 | 0.47 |
| pH | 9.42 | 0.25, 366 | 0.22 |
| PO2 | 1.00 | 1.00, 1.00 | 0.39 |
| PCO2 | 1.00 | 0.98, 1.02 | 0.92 |
| Lactate | 1.15 | 0.98, 1.34 | 0.09 |
| Creatinine | 1.11 | 0.84, 1.46 | 0.47 |
| ^1^OR = Odds Ratio, CI = Confidence Interval | | | |

Supplementary Table 7. Multivariate logistic model adjusted with covariates selected by uni-variable analyses for In-hospital mortality

| **Characteristic** | **OR**^1^ | **95% CI**^1^ | **p-value** |
| --- | --- | --- | --- |
| Group |  |  |  |
| T1 | 1.00 | Reference |  |
| T2 | 1.44 | 1.08, 1.86 | 0.04 |
| Weight | 0.97 | 0.96, 0.99 | <0.001 |
| SAPS_II | 1.03 | 1.00, 1.06 | 0.06 |
| SOFA_score | 1.23 | 1.10, 1.38 | <0.001 |
| Charlson_comorbidity_index | 0.99 | 0.88, 1.11 | 0.88 |
| MV_therapy |  |  |  |
| YES | 1.00 | Reference |  |
| NO | 0.78 | 0.30, 1.94 | 0.59 |
| Sedative_therapy |  |  |  |
| YES | 1.00 | Reference |  |
| NO | 1.38 | 0.59, 3.46 | 0.48 |
| Vasopressor_therapy |  |  |  |
| YES | 1.00 | Reference |  |
| NO | 1.66 | 0.86, 3.33 | 0.14 |
| T2DM |  |  |  |
| YES | 1.00 | Reference |  |
| NO | 1.41 | 0.73, 2.85 | 0.32 |
| Respiratory_rate | 1.04 | 1.00, 1.07 | 0.04 |
| Heart_rate | 1.01 | 1.00, 1.03 | 0.02 |
| WBC_count | 1.01 | 0.99, 1.04 | 0.28 |
| Hemoglobin | 0.91 | 0.80, 1.03 | 0.14 |
| pH | 8.93 | 0.42, 204 | 0.16 |
| Lactate | 1.10 | 0.94, 1.27 | 0.22 |
| Creatinine | 1.13 | 0.89, 1.43 | 0.3 |
| ^1^OR = Odds Ratio, CI = Confidence Interval | | | |

Supplementary Table 8. Multivariate logistic model adjusted with covariates selected by Boruta algorithm for In-hospital mortality

| **Characteristic** | **OR**^1^ | **95% CI**^1^ | **p-value** |
| --- | --- | --- | --- |
| Group |  |  |  |
| T1 | 1.00 | Reference |  |
| T2 | 1.45 | 1.10, 1.68 | 0.03 |
| Creatinine | 1.05 | 0.83, 1.31 | 0.69 |
| Platelet | 1.00 | 1.00, 1.00 | 0.03 |
| Charlson_comorbidity_index | 1.01 | 0.91, 1.11 | 0.88 |
| SOFA_score | 1.21 | 1.10, 1.33 | <0.001 |
| SAPS_II | 1.03 | 1.01, 1.06 | 0.02 |
| ^1^OR = Odds Ratio, CI = Confidence Interval | | | |
